# Greener Decellularization of Porcine Auricular Cartilage Using Supercritical Technology and Different Pretreatments for Application in Tissue Engineering

**DOI:** 10.1021/acsbiomaterials.4c02155

**Published:** 2025-06-19

**Authors:** Victor M. de Souza, Carolina C. Zuliani, Jéssica B. da Cunha, Juliana Carron, Carmen S. P. Lima, Ibsen B. Coimbra, Paulo T. V. Rosa, Ângela M. Moraes

**Affiliations:** † School of Chemical Engineering, 28132 University of Campinas − UNICAMP, 13083-852 Campinas, São Paulo, Brazil; ‡ School of Medical Sciences, University of Campinas − UNICAMP, 13083-887 Campinas, São Paulo, Brazil; § Institute of Chemistry, University of Campinas − UNICAMP, 13083-970 Campinas, São Paulo, Brazil

**Keywords:** decellularization, extraction, porcine auricular cartilage, supercritical fluid, osmotic shock, freeze−thaw, scaffold, tissue engineering

## Abstract

Scaffolds for tissue engineering can be obtained from synthetic or natural materials, with decellularized tissues being particularly attractive. Among these, porcine auricular cartilage is of special interest because of its availability, similarity to the human extracellular matrix (ECM), and cost-effectiveness. Decellularization of animal tissues yields extracellular matrices (ECM) rich in collagen, elastin, and glycosaminoglycans (GAGs), which are essential for providing mechanical support and creating a favorable environment for cell adhesion and tissue development. Traditional decellularization methods that rely on surfactants, such as sodium dodecyl sulfate (SDS), can have drawbacks, including protein denaturation, cytotoxic effects, the need for extensive washing, and the production of hazardous effluents. Alternative approaches involving the use of supercritical CO_2_ (scCO_2_) combined with cosolvents and preceded by specific tissue pretreatments have the potential to minimize ECM degradation, reduce effluent production, and allow for the recycling of CO_2_, thus lowering the overall carbon footprint. In this study, the decellularization of porcine auricular cartilage was investigated using osmotic shock and freeze–thaw pretreatments, followed by exposure to scCO_2_ combined with either butanol or ethanol. For comparison, traditional SDS decellularization was also performed. The decellularized tissues were assessed based on ECM structure, cell removal efficiency, and mechanical properties through histological analysis, DNA quantification, and mechanical compression testing. The results showed that none of the treatments fully decellularized the cartilage, likely due to the tissue’s high GAG content. However, the combination of freeze–thaw cycles followed by scCO_2_ treatment with butanol yielded the most favorable results, preserving the mechanical properties of the cartilage while minimizing ECM degradation.

## Graphical abstract

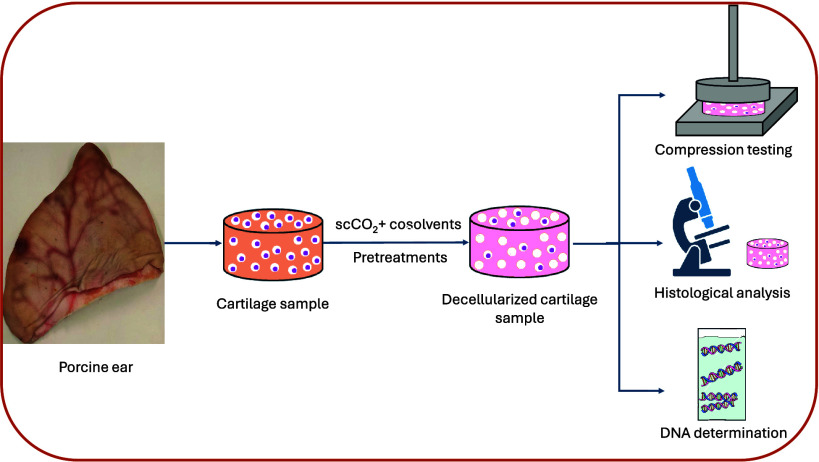

## 1 Introduction

Tissue and organ malfunctions are inherent consequences of diseases, accidents, or the natural aging process.^1^ Despite that biomaterials designed for interaction with biological systems in medical applications can be manufactured to produce functional replacements for damaged body parts,^2^ an emerging goal in this area is not merely on replacement but on the regeneration and repair of damaged tissues, a field known as tissue engineering.^3^

The fabrication of successful tissue engineering constructs relies on three fundamental components, known as the tissue engineering triad:^4^ the appropriate selection of cells, the presence of suitable signals, including biophysical cues and chemical mediators, and scaffolds for three-dimensional culture. The scaffolds should be capable of providing physical support for cell adhesion and growth while also mimicking the extracellular matrix (ECM). The ECM has primarily three classes of components: elastic fibers, made of elastin, important for tissue flexibility; collagen fibers, consisting of different collagen types, which improve mechanical strength and cell adhesion; and glycosaminoglycans (GAGs), which also provide cell adhesion sites and retain water, additionally assisting on the mechanical stability of the tissue.^5^

Various techniques are used to fabricate scaffolds, including casting, electrospinning,^6^ 3D printing,^7,8^ and bioprinting.^9^ Another procedure to fabricate scaffolds is the decellularization of allogenic or xenogenic tissues.^10^ This technique removes the cells from the ECM, which can then be used as scaffolds.^11^ The decellularized ECM has the necessary cues to cell adhesion, biologically compatible mechanical properties, and a similar composition to the human ECM, which are significant advantages when compared to synthetic scaffolds.^12^

Numerous commercially available biomaterials are made from decellularized ECM. Some prominent examples are Alloderm (Lifecell Corporations), made from decellularized human skin and used to treat skin wounds;^13^ Biodesign Dural Graft (Cook Biotech), derived from porcine intestinal mucosa, used for dura mater reconstruction;^14^ and MicroMatrix (Integra Life Sciences), produced from porcine bladder tissue and employed to treat skin burn wounds,^15^ among others. Clinical applications include the treatment of skin lesion,^16^ hernia surgeries,^17^ chest wall reconstruction,^18^ and orthodontic applications.^19^

Ideally, all cellular content must be removed from the tissue to ensure biocompatibility and minimize the risk of rejection.^20^ To evaluate the effectiveness of the decellularization process in removing cells, three primary criteria are typically applied: 1) no visible nuclear residues in the specimens, as observed through hematoxylin-eosin (HE) staining; 2) DNA content should not exceed 50 ng/mg of decellularized tissue; 3) residual DNA must not be longer than 50 base pairs. These criteria were established by Crapo et al.^21^ on the recent development of the technique, which focuses on DNA quantification, since it is the most internal component of cells, indicating the removal of other cellular content.^22^

Decellularization is usually performed with surfactants such as Triton X-100, Tween 80, or sodium dodecyl sulfate (SDS) to disrupt cell membranes, and using enzymes such as nucleases to degrade cell genetic material, facilitating the removal of cellular components from the tissue until only the ECM remains.^23^ However, surfactants and enzymes also damage the ECM and negatively affect its mechanical properties.^24^ Another disadvantage of surfactants is their cytotoxicity, which affects tissue recellularization, making extensive and expensive washing steps necessary to remove detergent residues.^25^ The generated effluent is an aqueous solution of cellular debris and surfactants, being both chemically and biologically hazardous, and its improper disposal may contaminate the environment.^26^ To reduce processing time, cost of manufacturing, and carbon footprint and to better preserve the ECM’s structure, technologies employing supercritical carbon dioxide (scCO_2_) for tissue decellularization have been explored in recent years.^24^

A substance reaches the supercritical state when its pressure and temperature exceed critical conditions. Supercritical carbon dioxide exhibits low viscosity, high density, and high diffusivity, making it an excellent apolar solvent.^27^ Carbon dioxide’s relatively mild critical conditions (73 atm and 31.1 °C)^28^ facilitate the use of scCO_2_ to process thermally sensitive materials. Upon pressure reduction, scCO_2_ reverts to a gaseous state, easily separating from the product, which enables its reuse as a solvent in subsequent cycles, thereby minimizing environmental impact compared to traditional solvents. Furthermore, scCO_2_ can be combined with polar cosolvents, such as ethanol or butanol, to enhance its effectiveness.^22^ Consequently, the application of scCO_2_ in decellularization processes is highly promising.^24^

Decellularization with scCO_2_ also results in microbial death,^29^ since scCO_2_ interacts with various cellular components, damaging organelle structures,^30^ promoting cell lysis, and inducing intracellular reactions that inactivate pathogenic microorganisms, even at low temperatures.^31^ Therefore, scCO_2_ may serve a dual purpose: as a decellularizing agent and as an effective sterilizing technique for biomaterials.^32^ This dual functionality may reduce the duration of dedicated sterilization steps, or completely remove its need, shortening the manufacturing process and thus making it more environmentally friendly.^33^

This technique does not remove cells as efficiently as surfactants, but cosolvents can enhance cellular removal.^24,34^ Durço et al.^35^ successfully decellularized cartilage tissue from chicken sternums, used as a model tissue, when employing scCO_2_ as a solvent and mixtures of scCO_2_ with ethanol, water, and butanol. The presence of cosolvents, especially butanol and ethanol, significantly increased the level of decellularization, although certain processing conditions also affected the ECM structure.

Some pretreatments can also disrupt cells before extraction with scCO_2_, facilitating the removal of cellular debris. Osmotic shock and freeze–thaw cycles are the most common pretreatments studied.^34,36^ In the pretreatment by osmotic shock, the tissue to be decellularized is continuously and alternately immersed in hypertonic and hypotonic solutions. This variance in osmotic pressure promotes cellular expansion and eventual rupture due to successive gains and losses of water, physically breaking the plasmatic membrane.^24^ This approach has shown promising results in bovine^37^ and porcine^38^ tissues. On the other hand, the freeze–thaw cycles consist of the samples’ continuous and alternating freezing and thawing. In this approach, ice crystal formation promotes the physical rupture of the cell membrane.^36,39^

In addition to choosing the appropriate decellularization method and pretreatment for biological material, it is critical to identify the most suitable tissue for a specific application.^24^ Ideally, the selected tissue should be sourced from a low-cost material. Porcine ear cartilage emerges as one of the prime candidates due to its high production in Brazil and relatively low market value. This tissue has a composition similar to the human ECM, containing collagen, elastin, and GAGs, which theoretically provide high biocompatibility and functionality, essential attributes for scaffolds.^40^

Decellularizing porcine ear cartilage can transform low-value byproducts from the food industry into high-value medical biomaterials. The potential of this approach is promising, as decellularization and reimplantation tests in pigs have shown high biocompatibility with the recipients.^40^

This work then focused on studying the decellularization process of porcine ear cartilage under supercritical conditions after different pretreatments, aiming for its future application as an implantable or graftable biomaterial for tissue reconstruction. Two distinct pretreatments (osmotic shock and freeze–thaw cycles) and three different decellularization strategies (scCO_2_ mixed with either butanol or ethanol, and SDS decellularization) were evaluated to determine the most efficient combination of methodologies. The effect of each technique on overall cell removal, ECM preservation, and mechanical compression resistance was assessed. Thus, the efficacy of seven distinct decellularization strategies was systematically analyzed on a single tissue type, still underexplored in the literature.

## 2 Materials and Methods

### 2.1 Material

The reagents used in this study were the following: butanol 99% (w/w) (Sigma-Aldrich), ethanol 99% (w/w) (Synth), CO_2_ 99.5% (White Martins), deionized water obtained from the Milli-Q system (Millipore), EDTA (Ecibra), histological paraffin (Synth), histological staining kit – picrosirius red and Masson’s trichrome (Easy Path), lithium chloride (Sigma-Aldrich), liquid nitrogen (White Martins), magnesium chloride (Synth), Mayer’s Hematoxylin (Sigma-Aldrich), phosphate-buffered saline solution (PBS, Sigma-Aldrich), proteinase K (Thermo Fisher), sodium dodecyl sulfate (Synth), sodium chloride (Synth), sucrose (Sigma-Aldrich), toluidine blue (Sigma-Aldrich), Tris-HCl (Synth), Triton X-100 (Synth), and xylene (Synth).

### 2.2 Cartilage Sample Collection

Frozen porcine ears were sourced from a local market, and samples were collected on the same day after thawing. The initial biological material was divided into three regions: proximal, medial, and distal, considering the positions from the region close to the cranium to the tip of the ear, as indicated in Figure 1A.

**Figure 1:**
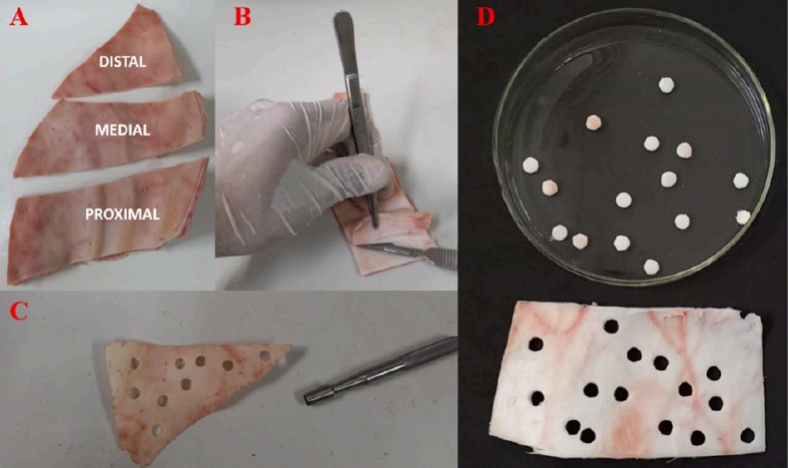
Ear region division and sample collection: A) Division in proximal, medial, and distal regions; B) Skin removal; C) Sample collection with punch; D) Collected samples.

The skin was removed with a scalpel (Figure 1B), leaving only the cartilage and some conjunctive tissue. Then, 6 mm cylindrical cartilage samples (Figure 1C and 1D) were collected with a stainless-steel punch (Rhosse, 6 mm). The diameter and thickness were measured with an analog caliper (Mitutoyo). The samples were weighed and stored in PBS at 5 °C.

To minimize tissue source variation, only samples weighing between 40 and 60 mg and 1.5 and 2.5 mm thick were used in the study. To prevent degradation, the decellularization protocols were started no longer than 24 h after the initial thawing of the tissues.

### 2.3 SDS Decellularization

The protocol utilized in this work was based on the method established by Funamoto et al.^41^ and modified by Casali et al.^22^ First, the samples were immersed in 10 mM Tris-HCl buffer (pH = 8) containing 0.2% (w/w) EDTA for 1 h at room temperature and 300 rpm mixing. This procedure was adopted to chelate Ca^2+^ ions, facilitating cell detachment.^5^

Then, all samples were transferred to 10 mM Tris-HCl buffer (pH = 8) with 0.1% (w/w) SDS for 48 h under the same mixing and temperature conditions. Afterward, the samples were rinsed with PBS (pH = 7.4) for 24 h at 300 rpm and room temperature (solution exchanged four times) to remove the excess SDS and cellular debris. Following, the mass, thickness, and diameter of the samples were determined. All specimens were stored in PBS at 5 °C.

### 2.4 Osmotic Shock Pretreatment

The osmotic shock protocol was based on the procedures described by Antons et al.^37^ and Lu et al.^42^ The samples were immersed in a solution of 3 M NaCl dissolved in PBS for 3 h at room temperature and 300 rpm. Then, the specimens were transferred to deionized water for 3 h under the same temperature and agitation conditions. This procedure was repeated three times, using the same saline solution but changing the deionized water at each cycle.

At the end of the three osmotic shock cycles, the samples were collected and stored in a PBS solution at 5 °C. Within 24 h, the specimens underwent treatment using supercritical fluid decellularization methods described in Section 2.6.

### 2.5 Freeze–Thaw Cycles

The freeze–thaw cycle procedure was based on the works of Antons et al.^37^ and Burk et al.,^43^ with small modifications. The samples were immersed in liquid nitrogen (−196 °C) for 2 min and then in deionized water for 10 min at 37 °C. This process was repeated six times, after which the samples were transferred to PBS solution and stored at 5 °C for up to 24 h before undergoing supercritical fluid decellularization.

### 2.6 Supercritical Fluid Decellularization

Supercritical fluid decellularization was conducted after sample pretreatment with osmotic shock or freeze–thaw cycles and on pristine cartilage for comparison. Ethanol 70% (v/v) and butanol 99% (w/w) were used as cosolvents. Figure 2 illustrates the supercritical decellularization apparatus used in the experiments. The procedure adopted was based on the works of Casali et al.^22^ and Durço et al.^35^

**Figure 2:**
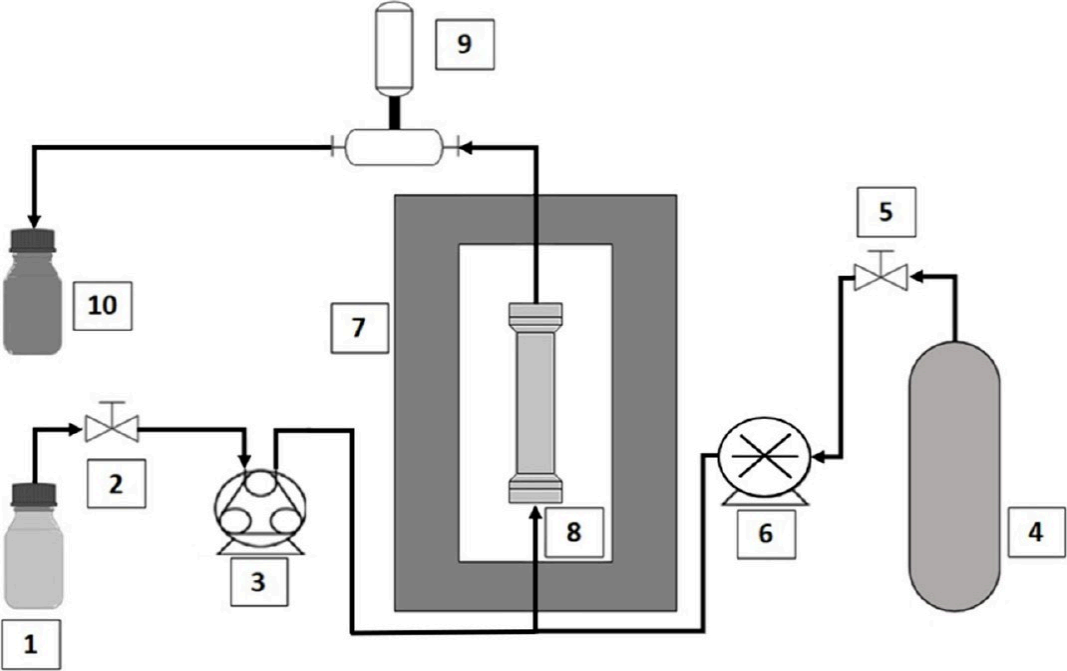
Supercritical decellularization system: 1) cosolvent reservoir; 2) valve; 3) HPLC pump; 4) CO_2_ cylinder; 5) valve; 6) CO_2_ syringe pump system; 7) oven; 8) decellularization vessel; 9) back pressure regulator (BPR) system; 10) residues reservoir.

Six samples were distributed inside the decellularization vessel (a 50 mL titanium column) separated by 3 mm glass spheres. The vessel was connected to CO_2_, cosolvent and exit lines and placed inside an oven (Nova Ética, Ethik EST. 420) to maintain the temperature at 37 °C. The CO_2_ exiting the cylinder was cooled to 5 °C to ensure it remained in a liquid phase and was pumped with a high-pressure syringe pump (DCI Systems, VPA) to the decellularization vessel until pressure increased to 270 bar. The pressure inside the vessel was maintained at 270 bar throughout the experiment with a back pressure regulator (BPR) system, consisting of a pressure-controlled syringe pump (Teledyne, ISCO Model 100DX) connected to the back pressure regulator valve (JBV). Any pressure increase opened the valve, resulting in the release of a mixture of CO_2_, cosolvent and cellular debris, thereby reducing the pressure. After the pressure release, the syringe pump closed the system, and the pressure was reestablished due to the continuous flow of CO_2_ inside the vessel. Therefore, a permanent pressure regime was established.

After pressure stabilization, the cosolvent flow was initiated by using a HPLC pump (Shimadzu LC 8A), and the procedure continued for 1 h. Then, the cosolvent flow was interrupted, and pure scCO_2_ was percolated through the decellularization vessel for 30 min to remove the excess cosolvent and continue to remove cellular debris. After this time, the CO_2_ flow was interrupted; the pressure was slowly decreased by changing the settings of the syringe pump used to control the BPR, and the decellularized samples were collected.

Slow depressurization of the system is essential, as sudden pressure release can produce temperature drops due to Joule-Thomson’s effect,^44^ causing ice formation in the tubes, clogging the apparatus, and damaging the pipeline and the pressure sensors. It can also cause bubble formation inside the samples, especially when the pressure is close to the critical point, leading to sample damage. For these reasons, in this study, the depressurization step lasted approximately 40 min, with a maximum pressure decrease of 5 bar/min under most pressure conditions and 1 bar/min when close to the critical point (between 50 and 90 bar). The BPR was also heated to 50 °C to enhance the system sealing and to avoid ice formation.

The cosolvent volumetric flow was set to 0.04 mL/min (equivalent to 0.032 g/min for ethanol 70% v/v and butanol 99% w/w),^28^ and the volumetric flow of CO_2_ was set to 1.96 mL/min. Considering the temperature and pressure of the decellularization vessel, CO_2_ density was 1.0274 g/mL, yielding a massic flow of 2.01 g/min.^28^ Given the temperature of 37 °C, the pressure of 270 bar, and the low percentage of cosolvent in the mixture, it can be assumed that the decellularization vessel was operated in a single, supercritical, phase.^28,44^

Seven different decellularization strategies were explored: one with SDS and six based on scCO_2_ combined or not with other approaches, as described in Table 1. Four scCO_2_ decellularization procedures were preceded by either osmotic shock or freeze–thaw cycles pretreatments and supercritical CO_2_ was mixed either with ethanol 70% (v/v) or butanol 99% (w/w) as cosolvent. In addition, a control group was established for each experiment without performing decellularization.

**Table 1:** Tested Decellularization Procedures

Acronym	Decellularization procedure
C	Control group: unprocessed
SDS	SDS decellularization
ET	scCO_2_ combined with ethanol 70% (v/v)
BT	scCO_2_ combined with butanol 99% (w/w)
OSET	Osmotic shock followed by scCO_2_ combined with ethanol 70% (v/v)
OSBT	Osmotic shock followed by scCO_2_ combined with butanol 99% (w/w)
FTET	Freeze–thaw followed by scCO_2_ combined with ethanol 70% (v/v)
FTBT	Freeze–thaw followed by scCO_2_ combined with butanol 99% (w/w)

After supercritical decellularization, all samples were weighed, analyzed regarding dimensions, and stored in PBS at 5 °C.

### 2.7 Histological Analysis

Samples from all treatments were stained with the histological stains described in Table 2 and examined within 24 h of the completion of decellularization using an optical microscope (Leica DM2500) with the aid of Leica Application Suite Software (version 4.6.2) to analyze cell removal and assess tissue damage. The specific targets for each staining procedure are also indicated in Table 2.

**Table 2:** Types of Cell Structures or Compounds Marked by Each Stain and the Colors Produced by Them

Stain	Cell structure or compound marked	Color observed
Hematoxylin-eosin (HE)	Cytoplasm and ECM	Pink/purple
	Cell nuclei	Blue/purple
Toluidine blue (TB)	Cell nuclei	Blue
	Gags	Purple
Masson’s trichrome (MT)	Collagen fibers	Blue
	Cell nuclei and elastin fibers	Black
Picrosirius red (PR)	Collagen fibers	Red
	Cell nuclei	Black

Basically, hematoxylin (H) stains nuclei in blue-purple, while eosin (E) binds to basic components in the cytoplasm and ECM, staining these regions pink,^45^ overall tissue structure and cell presence were analyzed using HE. MT and PR interact specifically with collagen fibers, an essential component of the ECM structure (MT stains collagen fibers in blue,^46^ while PR stains them in red^47^). TB binds to proteoglycans and was used to assess for the presence of GAGs, staining them in purple.^45^ It also stains cell nuclei in blue, complementing the analysis through HE images.^46^

### 2.8 Mechanical Compression Testing

Decellularization processes influence the mechanical properties of biological tissues due to ECM degradation resulting from the removal of essential components and dehydration.^22,24^ To evaluate the effect of different decellularization strategies on tissue resistance, compression testing of both decellularized and untreated materials were carried out using a texture analyzer (TA. XT. Plus, Stable Micro Systems). Four to five samples from each treatment were used to analyze the mechanical properties within 24 h of the completion of decellularization.

The samples were compressed to 80% at a strain rate of 0.05 mm/s to mimic conditions of severe stress that could lead to sample rupture and to minimize the effect of strain rate on mechanical properties values.^48^ The equipment was configured with a minimum force threshold of 0.1961 N to detect contact with the cylindrical probe and a break sensitivity of 0.04903 N. Maximum compressive stress (MCS), maximum deformation, and Young’s modulus were determined for each sample.

### 2.9 DNA Concentration Analysis

DNA was extracted using the technique described by Woodhead et al.,^49^ with lithium chloride (LiCl) and proteinase K. The samples were lysed in 1 mL lysis buffer (320 mM sucrose, 10 mM Tris-HCl pH 7.5, 5 mM MgCl_2_, and 1% Triton X-100). Subsequently, the concentrated cell pellet was resuspended in 400 μL of digestion buffer (10 mM Tris-HCl pH 7.5, 10 mM EDTA, 10 mM NaCl, 0.5% SDS, and proteinase K at 20 mg/mL), at 200 rpm and 55 °C for 48 h or until cell lysis was complete.

Then, 200 μL of 7.5 M LiCl was added to the samples, and they were incubated at −20 °C for at least 15 min. Afterward, the samples were centrifuged at 13,000 rpm for 10 min, and 1 mL of absolute ethanol at −5 °C was added to the supernatant. The precipitated DNA was centrifuged at 13,000 rpm for 10 min, washed with ethanol 70% (v/v) twice, and resuspended in TE buffer (2 M Tris base and 0.2 M EDTA, pH 8.0).

The samples were stored at −20 °C until the next step. DNA content was determined using a spectrophotometer (NanoDrop 2000, Thermo Scientific) at 260 nm.^22,50^

DNA concentration was measured in three groups: a control group (untreated samples); a group treated with SDS (as the most traditional decellularization method); and the group treated with scCO_2_ that showed the best preservation of mechanical properties, which was defined later in the study.

### 2.10 Statistical Analysis

Data statistical analysis was performed with the aid of the software Excel (version 2409 Build 16.0.18025.20160), applying Student’s and Tukey’s comparative tests with a significance level of 5%. Quantitative data were expressed in terms of mean values and standard deviation.

## 3 Results and Discussion

### 3.1 Characterization of Untreated Tissue

To assess the impact of each decellularization technique on the mechanical properties of the resulting tissues, it was first necessary to establish the relevant properties of the native porcine auricular cartilage tissue. The porcine ear was divided into three regions, and the samples were characterized regarding mass, thickness, and mechanical compression behavior. Five samples from each region were tested, with samples taken from three different ears.

The results are shown in Table 3. The tissue mass increased as the sample collection site approached the base of the head. Values of thickness, maximum compressive stress, and Young’s module of tissue samples from proximal and medial regions were not statistically different, but the values for the native biological material of the distal region are significantly lower.

**Table 3:** Properties of Different Regions of Non-Decellularized Porcine Auricular Cartilage Samples from Different Regions^a^

Region	Mass (mg)	Thickness (mm)	Maximum compressive stress (MPa)	Young’s module (MPa)
Proximal	75.1 ± 14.3^a^	2.43 ± 0.37^a^	8.96 ± 1.67^a^	18.88 ± 1.34^a^
Medial	58.3 ± 7.8^b^	2.01 ± 0.37^a^	9.40 ± 0.95^a^	16.75 ± 2.05^a^
Distal	36.8 ± 7.3^c^	1.23 ± 0.24^b^	6.85 ± 0.91^b^	11.57 ± 1.55^b^

a Different letters in the same column indicate significant difference at 95% confidence limits (Tukey’s test).

Sample harvesting from the proximal region was a rather complex procedure. The proximity to the animal’s head implies the presence of muscular groups that are difficult to separate from the cartilage without damaging the tissue of interest. Also, a high level of fat was observed in the tissue from this region. The excision of cartilage from the distal region was also challenging, mainly due to its low thickness. Therefore, tissue from the medial region was selected to continue the decellularization studies since it was easier to harvest the samples from it, and tissue from this region showed adequate property values, as observed in Table 3.

Despite selecting a single region for sample collection and using ears of similar shape and size from the same supplier, the properties still varied between ears, increasing the uncertainty in the analysis, as shown in Table 4. To better evaluate the impact of the treatments on such properties, each assay had its control group from samples collected from the same ear as the treated materials, on the same day.

**Table 4:** Mean Values and Variation Ranges of Different Properties of Tissue Collected from the Medial Region of Different Ears

Property	Mean value	Variation range
Thickness (mm)	2.0 ± 0.3	1.5–2.9
Diameter (mm)	5.9 ± 0.1	5.5–6.1
Mass (mg)	53.3 ± 8.9	37.5–73.0
Maximum compressive stress (MPa)	8.5 ± 1.5	5.2–12.1
Young’s modulus (MPa)	17.5 ± 2.4	12.7–22.8
Maximum deformation (%)	71.1 ± 7.8	57.1–80.0
Apparent density (mg/mm^3^)^a^	1.0 ± 0.2	0.6–1.3

a Defined as the ratio between the mass and volume of the samples.

### 3.2 Effects of Decellularization on Cell Removal and the Presence of GAGs

Cell removal was evaluated primarily through HE staining. As shown in Figure 3, all treatments resulted in tissue containing a residual nuclear material. Therefore, all the tested decellularization techniques should be improved to eliminate cellular content from the porcine ear cartilage entirely. Even the most traditional method (SDS exposure) led to genetic material retention in the tissue. HE staining revealed dark spots distributed throughout the cartilage tissues tested, attributed to cell lysis and dispersion of DNA within the tissue, which remains embedded in the cartilage matrix.

**Figure 3:**
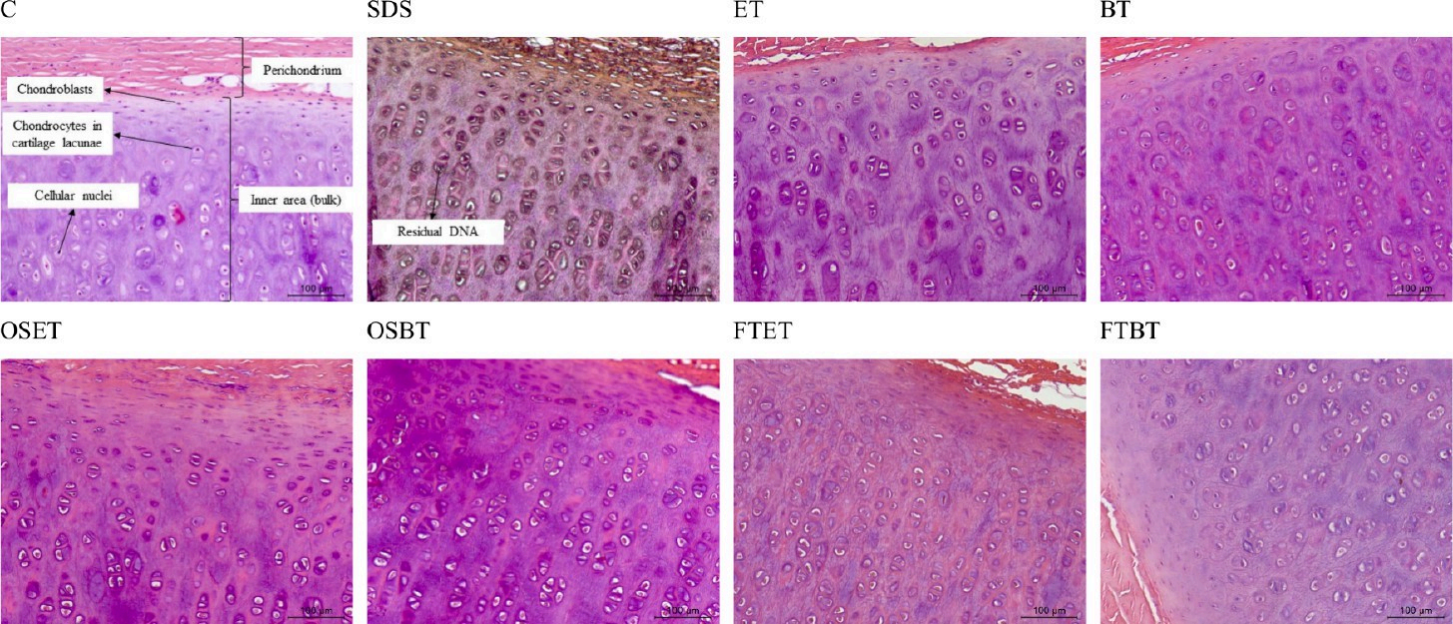
Histological analysis with hematoxylin-eosin (HE) for samples treated with different techniques: C) Non decellularized (control); SDS) SDS decellularization; ET) scCO_2_ + ethanol; BT) scCO_2_ + butanol; OSET) Osmotic shock + scCO_2_ + ethanol; OSBT) Osmotic shock + scCO_2_ + butanol; FTET) Freeze–thaw + scCO_2_ + ethanol; FTBT) Freeze–thaw + scCO_2_ + butanol.

The retention of DNA in the samples is due to the high compaction of the porcine auricular cartilage, which makes it difficult for scCO_2_ or SDS to penetrate the material and carry out the cellular debris.^40^ Another difficulty of cartilaginous tissue is that ECM composition varies with the position; pericellular coatings can be observed, which may increase mass-transfer resistance.^51^

GAGs have cell adhesion sites, which contribute to maintain the cells attached to the ECM, decreasing the decellularization efficiency.^52^ It is noticeable that all treatments maintained high proportions of GAGs, as observed with toluidine blue (TB) staining (Figure 4). The purple structures indicate a high concentration of GAGs, and the different tones observed were attributed to different light intensities in the microscope. The GAGs decrease decellularization efficiency but their presence in the treated tissue is highly desired, since they have widespread functions within the body, e.g. in cell signaling process, regulation of cell growth, proliferation, promotion of cell adhesion, anticoagulation, and wound repair.^53^

**Figure 4:**
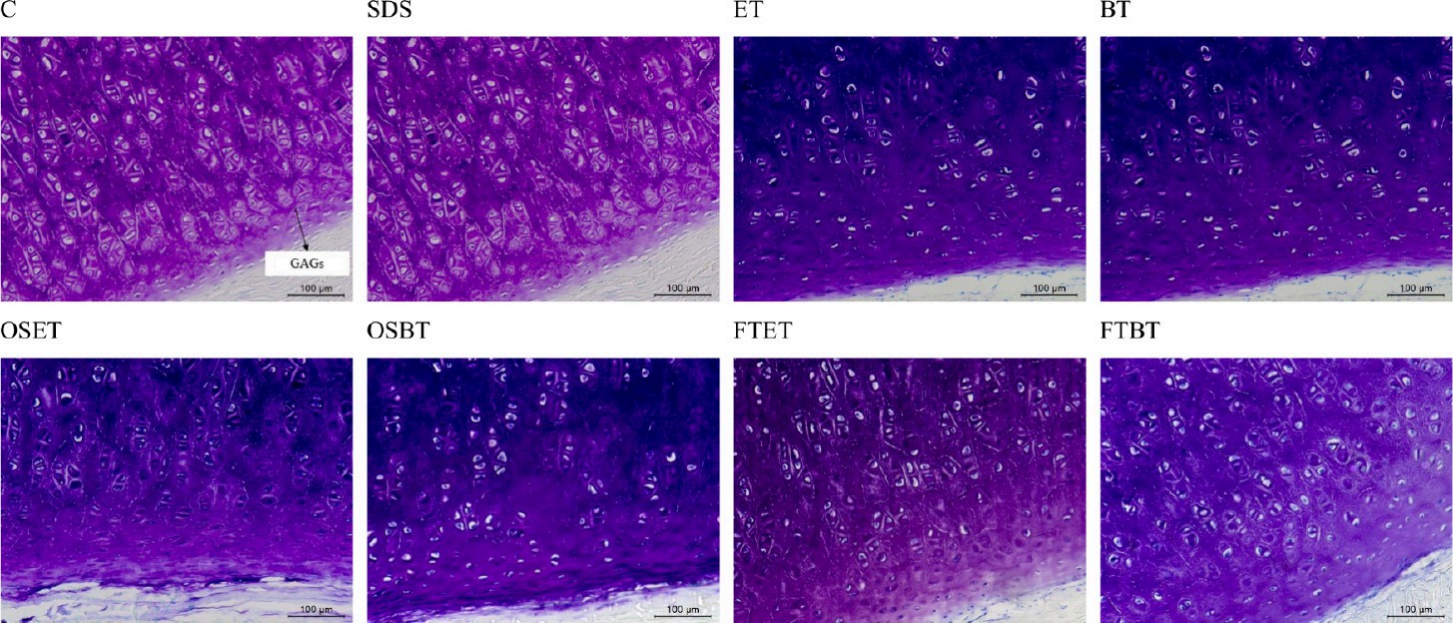
Histological analysis with toluidine blue (TB) for samples treated with different techniques: C) Non decellularized (control); SDS) SDS decellularization; ET) scCO_2_ + ethanol; BT) scCO_2_ + butanol; OSET) Osmotic shock + scCO_2_ + ethanol; OSBT) Osmotic shock + scCO_2_ + butanol; FTET) Freeze–thaw + scCO_2_ + ethanol; FTBT) Freeze–thaw + scCO_2_ + butanol.

### 3.3 Effects of Decellularization on the ECM Structure

Analysis of Masson’s trichrome (MT) staining provides essential insights into the effects of treatments on the ECM structure. MT staining results show that SDS treatment resulted in severe degradation of collagen fibers, as evidenced by the red color, both in the perichondrium and in the bulk of the cartilage regions, as shown in Figure 5. A more intense degradation is observed for the FTET treatment, resulting in a dark red color. In this case, the combination of the freeze–thaw cycles and the use of ethanol as cosolvent favored the ECM degradation, specially due to the freeze–thaw cycles, which may degrade collagen structure due to the rapidly formation of ice crystals, as described by Ding et al.,^54^ by Cao et al.^55^ and by Ozcelikkale et al.^56^ MT staining also shows elastic fibers, colored in black and preserved in all treatments.

**Figure 5:**
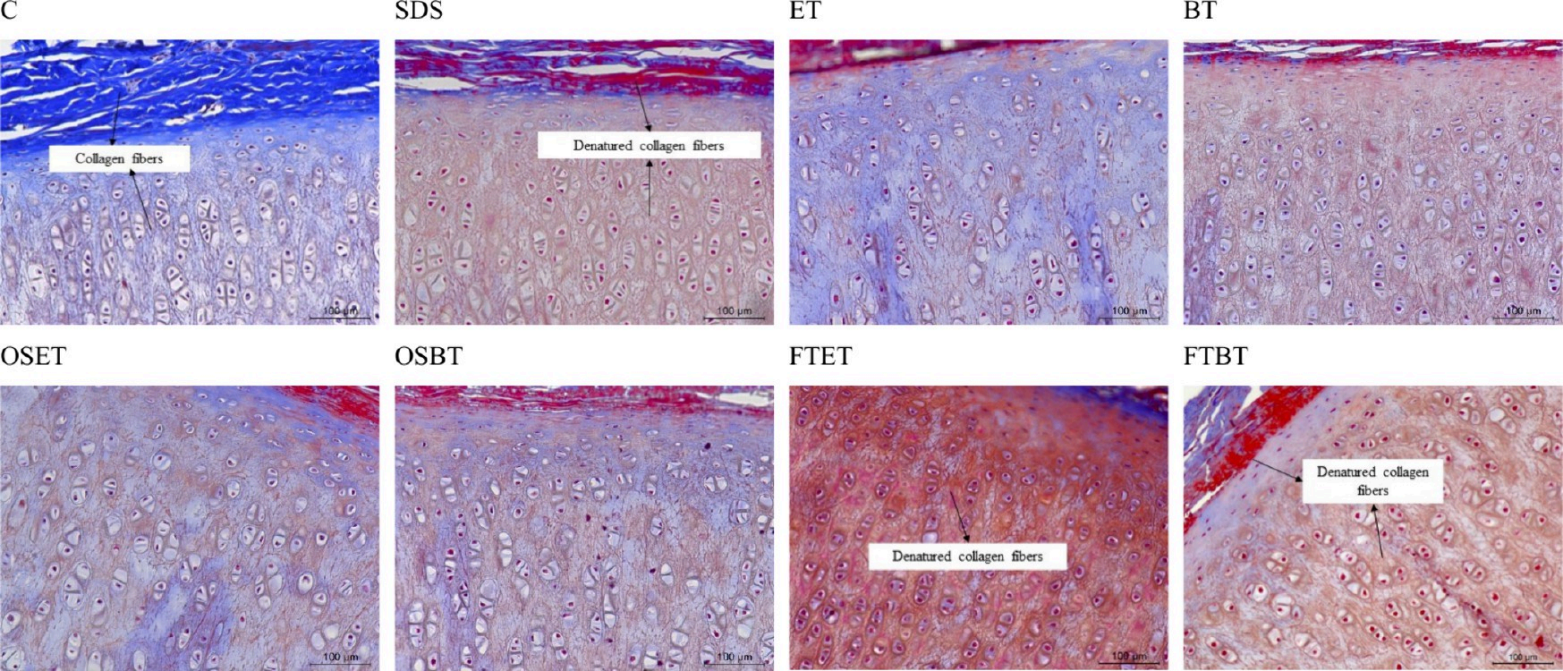
Histological analysis with Masson’s trichrome (MT) for samples treated with different techniques: C) Non decellularized (control); SDS) SDS decellularization; ET) scCO_2_ + ethanol; BT) scCO_2_ + butanol; OSET) Osmotic shock + scCO_2_ + ethanol; OSBT) Osmotic shock + scCO_2_ + butanol; FTET) Freeze–thaw + scCO_2_ + ethanol; FTBT) Freeze–thaw + scCO_2_ + butanol.

Samples treated with SDS treatment, when analyzed using picrosirius red (PR) staining (Figure 6), showed a darker red color, probably due to DNA spreading through the tissue, indicating cell lysis, as also observed with HE. When compared with the control, all treatments resulted in some damage to the collagen fibers. Nevertheless, similar to MT staining, PR staining revealed minimal differences among the various decellularization techniques.

**Figure 6:**
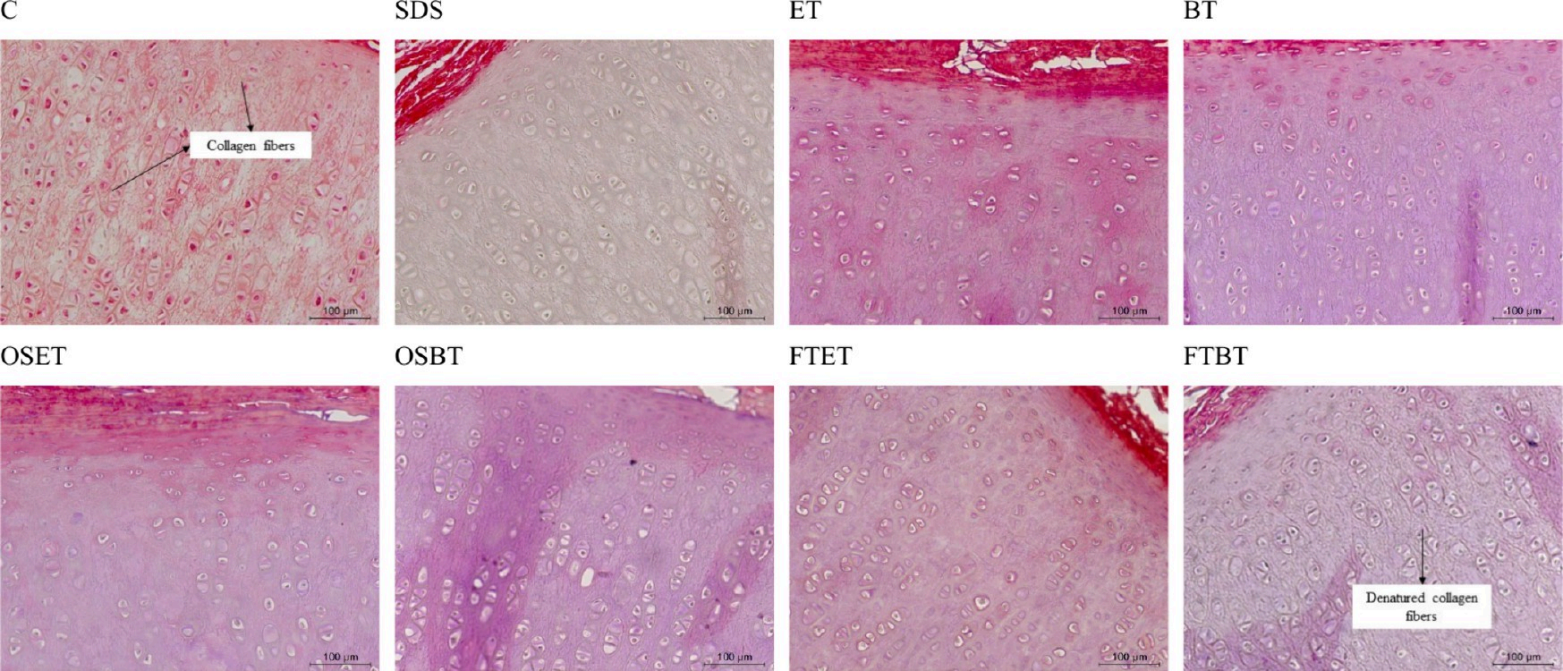
Histological analysis with picrosirius red (PR) for samples treated with different techniques: C) Non decellularized (control); SDS) SDS decellularization; ET) scCO_2_ + ethanol; BT) scCO_2_ + butanol; OSET) Osmotic shock + scCO_2_ + ethanol; OSBT) Osmotic shock + scCO_2_ + butanol; FTET) Freeze–thaw + scCO_2_ + ethanol; FTBT) Freeze–thaw + scCO_2_ + butanol.

### 3.4 Effect of Decellularization Treatments on Variation of Mass, Thickness, Diameter, Apparent Density, and Mechanical Properties of the Samples

The use of different decellularization strategies may not only lead to distinct results in terms of efficiency of cell removal but may also affect the mass, thickness, diameter, specific density, and mechanical properties of the samples in different ways, as shown in Figures 7 and 8.

Thickness was significantly affected only by treatments ET and OSET (Figure 7a), showing increased values in comparison to those of their respective control samples. Despite ECM dehydration^22^ may occur as a result of ethanol addition, leading to tissue shrinking, the denaturation of collagen fibers^57^ could have contributed to increase the biomaterial’s thickness. It is significantly increased in the OSET group, since the accumulation of sodium chloride might have increased the local osmotic pressure, leading to water accumulation. However, such an increase was not noticed in samples’ diameter as a result of any treatment (Figure 7b). A probable explanation may also involve collagen fiber orientation, disfavoring radial expansion with fluid accumulation.^3^

**Figure 7:**
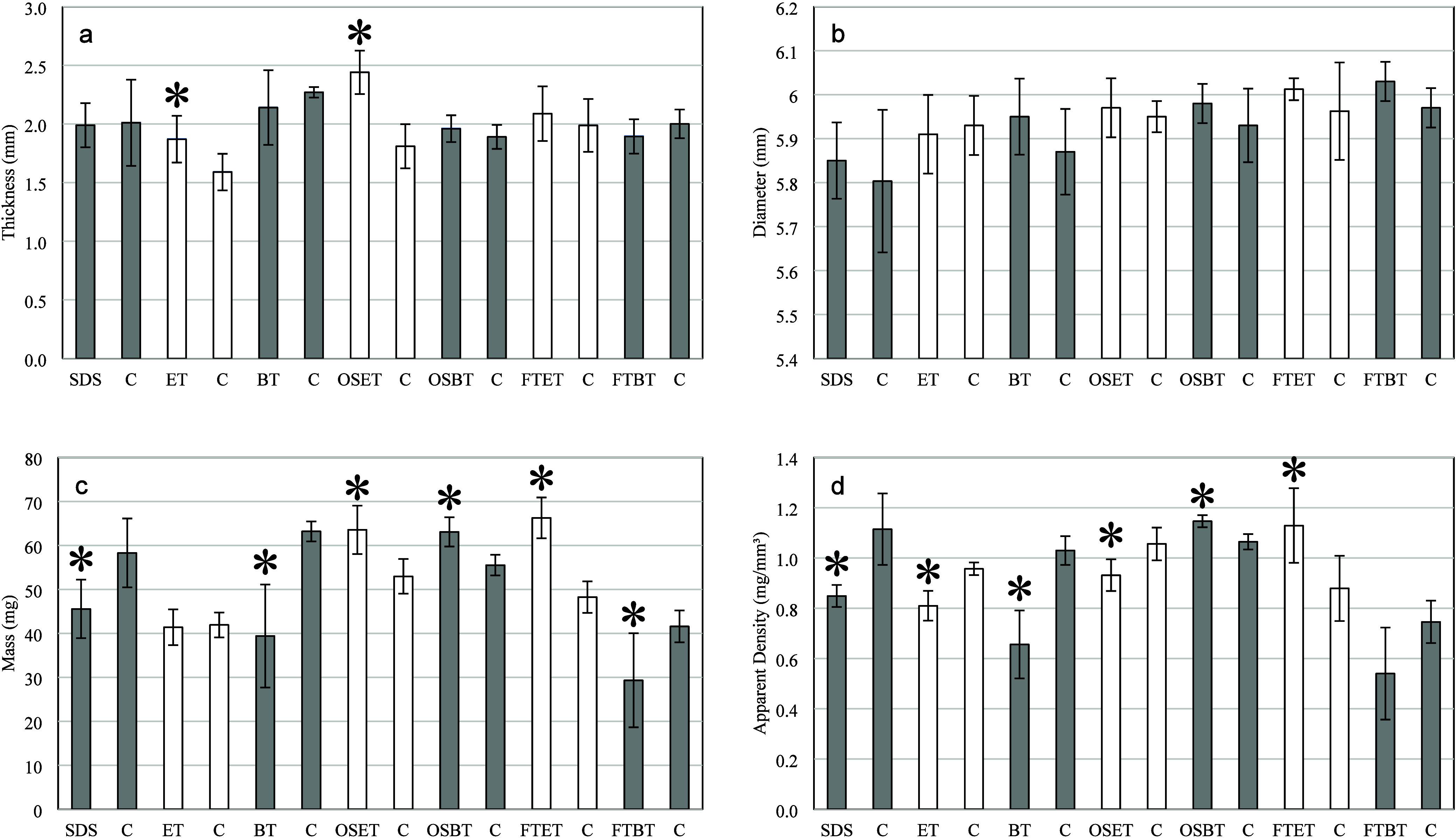
Effect of the different decellularization strategies on the average thickness, diameter, mass, and apparent density of the samples. The symbol * indicates a statistically significant difference between treated samples and the corresponding control group through Student’s test (*p* < 0.05).

As indicated in Figure 7c, all treatments, except for ET, affected the samples mass, probably due to inefficient cell removal by this method, as pointed out by other works.^21,22,24^ The techniques involving osmotic shock (OSET and OSBT) lead to an increase in the mass, probably due to the accumulation of ions during the pretreatment, increasing, as a consequence, water retention.^58^ The FTET approach also increased the mass of the tissues, probably due to the denaturation of collagen: this process may increase the number of available hydroxyl groups in the molecule, increasing water retention.^54,57^ All remaining techniques caused a decrease in mass due to the removal of cells, water, and ECM components.^36^

All treatments affected the apparent density except for FTBT (Figure 7d). If the treatment washed out ECM components, a higher weight loss would be expected, which could lead to a lower apparent density, as observed for samples treated with SDS, BT and OSET. Therefore, it is coherent to assume that the FTBT treatment maintained most of the ECM components and that the mass reduction is due to dehydration.

According to the results shown in Figure 8a, the reduction in maximum compressive force was statistically significant for samples treated with OSET, OSBT, and FTET possibly due to the denaturation of collagen fibers and removal of ECM components,^36^ which are essential for mechanical stabilization during compression.^5,48^ The remaining treatments did not result in statistically significant variations.

**Figure 8:**
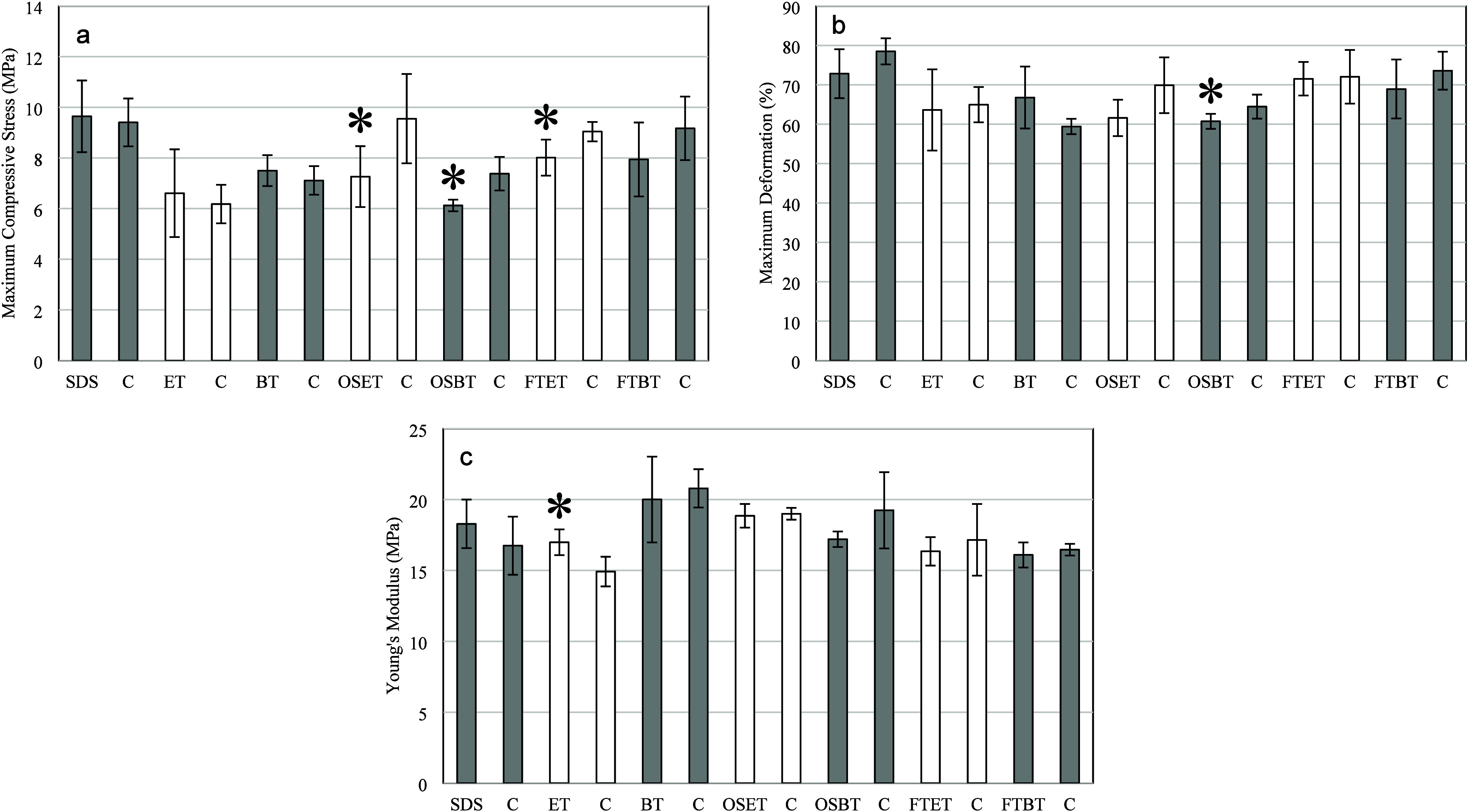
Effect of the different decellularization strategies on the mechanical properties of the cartilage tissue. The symbol * indicates a statistically significant difference between treated samples and the corresponding control group through Student’s test (*p* < 0.05).

Agreeing with the results attained in the analysis of maximum compressive force (Figure 8b), the treatment OSBT decreased the maximum deformation because it probably removed or denatured ECM components.^11^ The other decellularization conditions did not significantly affect this property.

The only treatment that affected Young’s modulus was scCO_2_ and ethanol at 70% (Figure 8c), which increased the elasticity modulus when compared to the control group probably due to collagen denaturation and removal of other structural components of ECM.^24,57^

Qualitative key observations from the characterization of the decellularized materials using the different approaches are summarized in Table 5, with the expected and/or preferred result for each analysis highlighted in gray.

**Table 5:** Comparison of Effects of Each Decellularization Technique on Different Tissue Properties^a^

Strategy	Thickness (mm^3^)	Diameter (mm^3^)	Mass (mg)	Apparent density (mg/mm^3^)	Maximum compressive stress (MPa)	Maximum strain (%)	Young’s modulus (MPa)
SDS	NA	NA	-	-	NA	NA	NA
ET	+	NA	NA	-	NA	NA	+
BT	NA	NA	*--	*--	NA	NA	NA
OSET	++	NA	+	-	-	NA	NA
OSBT	NA	NA	+	+	-	-	NA
FTET	NA	NA	++	+	-	NA	NA
FTBT	NA	NA	-	NA	NA	NA	NA

a Variation levels: a) for increased values: + ++: high (from 67 to 100%); + +: medium (from 34 to 66%); + : low (up to 33%); b) for reduced values: *---*: high (from 67 to 100%); *-- : medium (from 34 to 66%); -: low (up to 33%); NA: not altered.

Ideally, structural components should remain intact in a decellularization process, minimizing variations in thickness, diameter, maximum compressive stress, maximum deformation, and Young’s modulus.

As discussed previously, a decrease in the mass would be expected. Increases in mass may indicate the undesirable presence of compounds used during the decellularization process that are not efficiently reduced. However, they can also indicate increased water retention by the treated material, which may be desirable. A reduction in the apparent density may point to the loss of ECM structural components or to an increase in the sample volume. However, if the dimensions of the material reduce after decellularization, this may also contribute to increasing the apparent density.

In summary, taking all of the considerations into account, decellularization of porcine auricular cartilage using freeze–thaw cycles followed by treatment with supercritical CO_2_ and butanol (FTBT) resulted in a better-preserved material with minimal variation in mechanical properties. For this reason, this method was considered the most effective treatment among the seven procedures tested.

### 3.5 DNA Analysis

The DNA concentration was measured in the control group, the SDS-treated samples, and the FTBT samples since this method yielded the best results in mechanical properties. The results achieved are described in Table 6.

**Table 6:** DNA Concentration on Samples from Selected Groups^a^

Sample group	DNA concentration (ng/mg)
Unprocessed (C)	13.1 ± 0.6 ^a^
SDS decellularization (SDS)	09.0 ± 1.3 ^a^
Freeze–thaw+scCO_2_+butanol (FTBT)	08.7 ± 3.8 ^a^

a Different letters in the same column indicate significant difference at 95% confidence limits (Tukey’s test).

As anticipated, a higher concentration of DNA is observed in the unprocessed tissue. SDS and FTBT reduce DNA concentration in similar proportions, although no statistically significant difference between the groups is observed. Despite the HE staining results demonstrating presence of nuclear material within the tissue, the DNA concentration across all samples remained below 50 ng/mg, consistent with the criteria established by Crapo et al.^21^ for effectiveness of decellularization.

Cartilaginous tissues inherently shows fewer cells than other tissues, and as a consequence, have reduced DNA content.^59^ Therefore, since porcine auricular cartilage meets the low DNA concentration criteria even before exposure to decellularization processes, additional criteria beyond DNA content may be required to assess the treatment efficacy for this type of tissue and its application as a scaffold material in tissue engineering. The determination of phospholipid concentration could improve the evaluation of decellularization processes since it is an important component of cellular membranes. Another possible improvement would be the evaluation of the collagen and elastin integrity through immunohistochemical analysis.

## 4 Conclusions

The medial region of the porcine ear can be considered the most suitable for scaffold fabrication due to its ease of sectioning from other tissues and to its adequate properties.

Histological analysis and DNA quantification indicate that the decellularization of porcine auricular cartilage using supercritical fluids and associated pretreatments alone does not result in complete tissue decellularization, a result that was similarly unattainable with SDS treatment. Assessing DNA integrity could provide insight into whether the DNA was structurally compromised but not removed or remained entirely unaffected by the decellularization process.

None of the decellularization strategies significantly removed GAGs, which play a key role in cell adhesion and other important biological functions. The retention of GAGs is highly desirable for applications in tissue engineering; however, these molecules, along with the significant compactness of porcine auricular cartilage, may impair the complete cell removal during decellularization. To better evaluate this effect, further investigation of the GAG concentration in the tissue should be conducted.

The SDS treatment significantly reduced the mass and the apparent density of the tissue samples, also denaturing ECM components (mainly collagen) and resulting in a wide dispersion of DNA throughout the biomaterial. Decellularization strategies involving osmotic shock resulted in increased mass, persistence of cells, and collagen fiber denaturation within the ECM.

In contrast, the outcomes of decellularization strategies incorporating freeze–thaw cycles as a pretreatment varied depending on the cosolvent. When scCO_2_ and ethanol were used, pronounced collagen fiber denaturation was evidenced by Masson’s trichrome staining. Conversely, butanol as a cosolvent produced no significant changes in variables other than mass and apparent density, which were attributed to loss of moisture and cellular components. While collagen denaturation was detected in the butanol-treated samples, it was considerably less severe compared to that induced by ethanol.

Strategies employing freeze–thaw cycles as pretreatment also yielded different results depending on the cosolvent. The combination of freeze–thawing with scCO_2_ and ethanol resulted in severe denaturation of ECM collagen fibers, as demonstrated by Masson’s trichrome staining. In contrast, the process involving butanol as a cosolvent did not affect any variable, except for mass. Collagen denaturation was observed but was much less significant than that observed in the presence of ethanol. Immunohistochemical analysis of the cartilage could provide a better understanding of structural damage of the ECM structures.

Decellularization of porcine auricular cartilage through freeze–thaw cycles, followed by treatment with scCO_2_ and butanol, produced the most preserved biomaterial, which exhibited minimal variation in mechanical properties. Future studies should focus on optimizing this technique to enhance cell removal, to develop a more efficient and environmentally sustainable decellularization process.

## Data Availability

Supporting data will be made available on request.
